# Geochemical fractions of rare earth elements in soil around a mine tailing in Baotou, China

**DOI:** 10.1038/srep12483

**Published:** 2015-07-22

**Authors:** Lingqing Wang, Tao Liang

**Affiliations:** 1 Key Laboratory of Land Surface Pattern and Simulation, Institute of Geographical Sciences and Natural Resources Research, Chinese Academy of Sciences, Beijing 100101, China

## Abstract

Rare earth mine tailing dumps are environmental hazards because tailing easily leaches and erodes by water and wind. To assess the influence of mine tailing on the geochemical behavior of rare earth elements (REEs) in soil, sixty-seven surface soil samples and three soil profile samples were collected from different locations near China’s largest rare earth mine tailing. The total concentration of REEs in surface soils ranged from 156 to 5.65 × 10^4^ mg·kg^−1^ with an average value of 4.67 × 10^3^ mg·kg^−1^, which was significantly higher than the average value in China (181 mg·kg^−1^). We found obvious fractionation of both light and heavy REEs, which was supported by the North American Shale Composite (NASC) and the Post-Archean Average Australian Shale (PAAS) normalized concentration ratios calculated for selected elements (La_*N*_/Yb_*N*_, La_*N*_/Sm_*N*_ and Gd_*N*_/Yb_*N*_). A slightly positive Ce anomaly and a negative Eu anomaly were also found. For all 14 REEs in soils, enrichment was intensified by the mine tailing sources and influenced by the prevailing wind.

Rare earth elements (REEs) include the lanthanides, the elements with atomic numbers 57–71. Because scandium and yttrium exhibit similar properties to the lanthanide family, they are also considered rare earth elements^1^. REEs can be further divided into two groups: the light rare earth elements (LREEs) and the heavy rare earth elements (HREEs). LREEs are the lower atomic weight elements, lanthanum to europium, while HREEs are gadolinium to lutetium and yttrium. REEs are highly electropositive and are predominantly trivalent (Ln^3+^), with the exception of cerium (Ce^4+^) and europium (Eu^2+^) in some environments. Since most REEs possess similar atomic radii and oxidation states, REEs can substitute for each in various crystal lattices. This capability of substitution leads to multiple REE occurrences within a single mineral and has resulted in a wide distribution within the Earth’s crust^2^. REEs are found in a wide range of mineral types, including halides, carbonates, oxides, phosphates and silicates. The abundance of REEs within Earth’s crust varies widely across individual REEs, ranging from the most abundant at 66 ppm of cerium (exceeding other important metals including copper −27 ppm and lead −11 ppm) to 0.28 ppm for thulium^3^,^4^.

Despite vast global distribution, REEs are mainly mined in China. In 2011, China produced over 90% of the world’s rare earth supply while owning only 23% of the world’s total reserves^5^. Due to the increase of rare earth mineral mining, China’s rare earth reserve has drastically decreased from 75% in 1970 to 23% in 2011^5^,^6^. The surface mining and heap leaching of China’s unique ion-adsorption rare earth resources have caused severe environmental damage, such as soil erosion, pollution, and acidification. Moreover, REEs have long been used in China as additives in fertilizers and as growth promoters in livestock feed. The long-term effects and damage of such uncontrolled discharge of REE-based chemicals into the environment remain to be determined.

REEs have been characterized neither as essential elements for life nor as strongly toxic elements in the environment^7^. Although the environmental toxicity of REEs is largely unknown, environmental contamination has already been found in some mineralized areas as well as soils that are affected by the long-term application of sludge. Several negative effects of REEs on organisms have been reported. For example, Waring and Watling (1990)^8^ and Pairon *et al.* (1995)^9^ have documented that some inhaled REEs tend to accumulate in human lung and lymph nodes. However, the mobility of REEs and their possible impacts on ecosystems are still relatively unknown and thus, potential risks for human health and environment cannot be currently estimated. Therefore, it is important to explore the environmental presence of REEs and properly predict the potential deleterious effects.

Bayan Obo ore deposit in the Inner Mongolia region of North China is the world’s largest rare earth ore deposit accounting for 59.3% of the world’s rare earth reserves^10^. The amount of tailing and slag is constantly increasing from the processes of mining, dressing, smelting and separating of rare earth. The Bayan Obo mine produces approximately 8.0 million tons of tailings each year^11^. Most of this enormous waste is typically disposed of in open dumps. Tailing is one of the major sources of REE environmental pollution since the tailing is powdery (and thus, potentially mobile) and its quantity is larger than slag. In this study, we investigated REEs distribution in the soil around the dumped tailing to assess pollution levels. The results presented herein provide scientific evidence of the ecological risk associated with the excavation of rare earth minerals and advocate the prevention of REE pollution in soil and eco-resumption.

## Materials and methods

### Study area

Baotou (40°14′56″–42°43′49″ N, 109°15′12″–111°26′25″ E) is located in the west of Inner Mongolia, which has become the largest rare earth production hub in China since the discovery of the giant Bayan Obo deposit in 1927^12^. This area has a temperate continental climate with an average annual temperature of 6.5 °C. The average annual precipitation is about 240–00 mm, with an evaporation of 1938–2342 mm. Due to the aridity and elevation, the temperature differences between day and night can be large, especially in spring. Strong winds occur most frequently in spring and winter. The prevailing wind direction is northwest, and the average wind speed is 3 m/s. The average number of days annually with strong wind, floating dust, and dust storms are about 46 days, 25.9 days and 43.3 days per year, respectively.

The Baotou REE tailing dam, located 12 km to the west of Baotou City and owned by the Baotou Iron and Steel Corporation, produces approximately 8.0 million tones of tailing each year^11^,^13^. These tailing powders are usually discharged into the open dumps through open slots by circulating water. Covering an area of 12 km^2^, with about 3.5 km from south to north and 3.2 km from east to west, the dam has become the largest rare earth tailing reservoir in the world since the use began in 1965. Since the tailing is powdery and its quantity is large, it is one of the major sources for environmental pollution.

The chemical compositions and mineral compositions of the Baotou tailing are listed in Table 1. The tailing is composed of various mineral matters, such as calcium, silicon, iron, rare earth compounds and fluorite. The dam now stores a large quantity of rare earth minerals discarded from the flotation-hydrometallurgical processing of Bayan Obo ore. The average grade of REEs in the tailings has increased from 6.8% of raw Bayan Obo ore to 8.85%^12^. It is estimated that about 9.3 × 10^6^ tons of the rare earth tailings of Bayan Obo have been piled up in the tailings dam^14^. Part of the tailing area is exposed to air and thus, under strong winds, a large amount of tailing powders with high REE concentrations can contaminate the surrounding environment potentially, leading to severe environmental deterioration.

### Sampling and measurement

**Figure 1:**
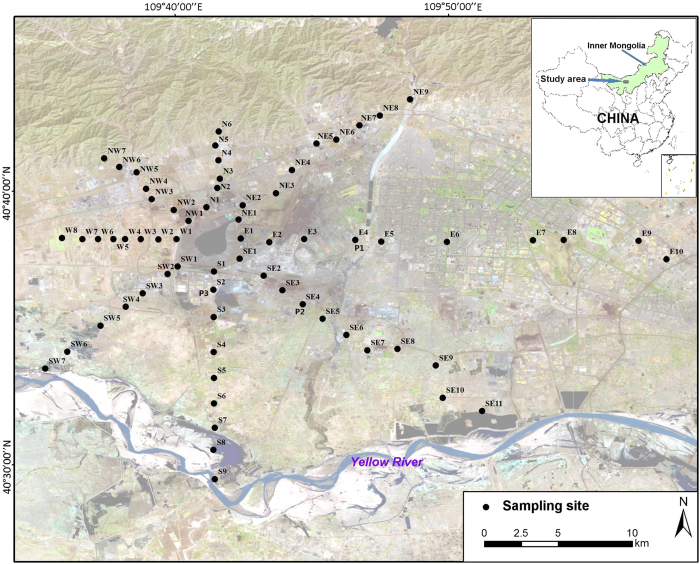
Map of the study area and sampling sites (ArcGIS, version 10.02).

A total of sixty-seven surface soil samples were collected at approximately a 0–10 cm depth at locations around the Baotou rare earth mine tailing. At each point, five samples were collected and uniformly mixed. According to the position of mine tailing and the prevailing wind direction, these samples were collected from areas in different directions. The overall coverage of sampling area was nearly 826.2 km^2^. The coordinates of the sample locations, as shown in Fig. 1, were recorded with a portable GPS. The main soil type in this district is chestnut soil (Haplic Krastazem, FAO) whose basic properties are shown in Table 2. Three soil profile samples (labeled as Pn, n = 1, 2 and 3), collected at a depth as much as 70 cm, were also selected to observe the vertical distribution of REEs. A total of 11 soil samples at depths of 0–3, 3–6, 6–9, 9–12, 12–15, 15–20, 20–25, 25–30, 30–40, 40–50, and 50–70 cm were taken for every soil profile. After collection, all samples were immediately stored in a portable cryostat and transported to the laboratory.

The collected soil samples were freeze-dried and then sieved through a 2 mm polyethylene sieve to remove plant debris, pebbles, and stones. Afterwards, the samples were thoroughly mixed, ground, and sifted by a 100-mesh sieve for subsequent geochemical analysis. Soil particle size was determined by a Mastersizer 2000 Type Laser Particle Analyzer (Malvern Instruments, Malvern, England). Soil pH value was measured with a glass electrode in a 1:2.5 soil/water suspension using a 1 M CaCl_2_ solution. Available phosphorus was measured using a sodium bicarbonate (0.5 M NaHCO_3_, pH 8.5)^15^, and soil organic matter was determined based on the loss in ignition method^16^.

To measure REEs, soil samples were digested with HNO_3_–HF–HClO_4_ and analyzed by inductively coupled plasma-optical emission spectrometry (ICP-OES, OPTIMA 5300DV, Perkin Elmer)^16^. The detection limit of ICP-OES is 10 μg·L^−1^. All measurements were carried out in duplicate. Quality control was maintained with certified reference samples GBW07303 from the National Research Center for Certified Reference Materials (Beijing, China). The readings for the reference samples were within 5% of the reported values. Calculations were made using MS Excel and for statistical analysis, SPSS 13.0 was used. ORIGIN 8.0 was used for creating figures. A global positioning system (GPS) was used to record the locations of the samples, and the geostatistical analysis was carried out with the extension Geostatistical Analyst of the GIS software ArcGIS (version 10.02).

Enrichment factor (EF) is widely used to estimate the anthropogenic impact on soil. EF is based on the normalization of analytical data against the reference element and is defined by the following formula^17^,^18^,^19^:


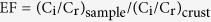


where C_i_ and C_r_ are the concentrations of the considered element and the reference element, respectively, in either the sample or Earth’s crust. In theory, a reference element should not be influenced by anthropogenic activities and should not be affected greatly by weathering processes. The most commonly used reference elements are Al, Ca, Fe, Li, Mn, Sc, and Sr^17^,^18^. In this study, Al was used as a conservative element to calculate the EFs of REEs. Calculations were made using content values of REEs and Al in the upper continental crust from Wedepohl (1995)^20^. In general, an EF < 1 indicates depletion and an EF > 1 indicates enrichment of the element considered. Soil samples can then be given a contamination category based on the enrichment factor.

## Results and discussion

### REEs concentrations in soil

The concentrations of REEs in the soils near the tailing as well as concentrations of REEs in the upper continental crust^3^ and the background values in this region are presented in Table 3,^21^. A summary of the main statistical parameters (mean, median, range, and standard deviation) was also shown. All the data were analyzed by the Kolmogorov-Smirnov (K-S) test for normal distribution. The results of the K-S test (*p *< 0.05) indicated that all REE concentrations were not normally distributed. As a result, the geometric mean or the transformed mean (log transformed or Box-Cox transformed) were used to describe the average concentrations^22^. After a box-cox transformation, the results successfully passed the K-S test for normality.

The coefficient of variation (CV) can be used to compare in relative terms the variability of the same property under similar values of variances and different means. Low CV values correspond to a spatially homogeneous distribution of REE concentrations, whereas high CV values indicate a non-homogenous surface distribution in the study area^23^. The CVs of all REEs, except Er, Yb and Lu, were relatively high, greater than 100%, indicating a high variability. The spatial variability of Er, Yb and Lu, however, was moderate with the CVs only fluctuating from 33.1% to 89.1%.

The concentration of REEs in the soil samples ranged from 156 to 5.65 × 10^4^ mg·kg^−1^ with a mean value of 4.67 × 10^3^ mg·kg^−1^. Thus, the measured mean REEs levels in the soil samples were much higher than the background values of REEs in soil of Baotou region^21^. Result observed for the sum of all REEs (∑REE) in the present study was also much higher than that reported by Hu *et al.*^7^ who found a mean REE concentration of 181 mg·kg^−1^ (1225 soil samples). Others have found slightly lower concentrations of REEs in Japan (98 mg·kg^−1^)^24^, Australia (105 mg·kg^−1^)^25^ and Germany (305 mg·kg^−1^)^26^.

The LREE/HREE ratio ranged largely from 5.05 to 34.2 with an average of 12. The LREE/HREE ratio showed that the content of LREE is significantly higher than that of HREE. The content of LREEs accounted for 90.9–99.6% of the total REEs content in the investigated soils. This percentage is in agreement with the trend that was observed in the Bayan Obo ores, and thus indicates that the concentrations and distributions of REEs are influenced by the rare earth mineral mining activities. The concentrations of individual REEs tend to decrease with increasing atomic number, and REEs with even atomic numbers are more frequent than their neighbors with odd atomic numbers, according to the Oddo–Harkins rule^12^,^27^: Ce > La > Nd > Pr > Sm > Gd > Dy > Er > Yb > Eu > Tb > Ho > Tm > Lu. REE concentrations are in the same order of magnitude as that in the earth’s crust as described by Taylor and McLennan (1995)^3^, similar to that in Bayan Obo ores in 2012^28^.

The correlation analysis between REEs in the soil samples showed that there is a statistically significant correlation (p < 0.05) between all the elements examined (Table 4). These findings confirm the aforementioned results and provide evidence for similar input sources from tailing and common geochemical characteristics of the elements. Since these elements in soil are limitedly mobile, REEs can continuously accumulate in surface soil via various pathways such as atmospheric deposition^1^, mining activities^29^ and application of REE fertilizers^30^. Results in the present study confirmed that soil environment near the tailing area was devastatingly polluted from many years of the mine tailing.

### Spatial distribution of REEs in surface soils

Semivariograms of the REE concentrations were calculated and fitted to a spherical model. The variogram model parameters are listed in Table 5. The ratio of the nugget effect (C_0_) over sill (C_0_ + C), r = C_0_/(C_0_ + C), expresses spatial correlations of REEs and provides a quantitative basis for interpolating unsampled locations. If the ratio was less than 25%, the REE concentrations were considered strongly spatially dependent, between 25–75%, moderately spatially dependent, and greater than 75%, weakly spatially dependent^31^. The ratio of all REEs was found to be less than 25%, and therefore indicating a strong spatial dependence.

**Figure 2:**
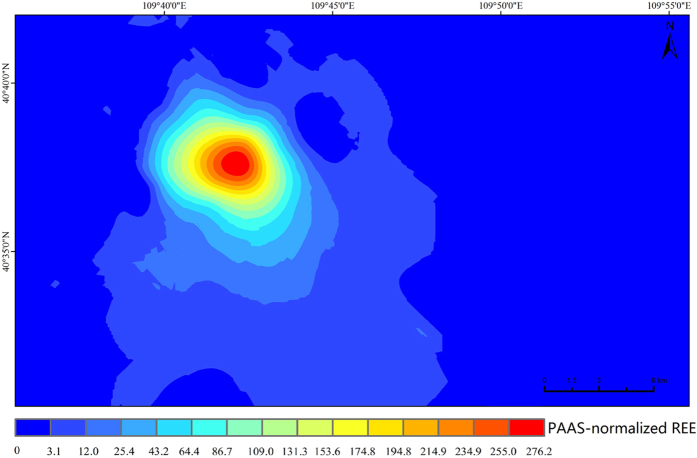
Estimated map of REEs concentration in surface soils normalized to PAAS (ArcGIS, version 10.02).

The spatial distribution of REE concentrations is useful to assess the possible sources of enrichment and to identify areas with high REE concentrations. The spatial distribution map of the REE concentrations in soil normalized to PAAS (Post Archaean Australian shale)^32^ is shown in Fig. 2. The concentrations of REEs in the studied area exhibit considerable variation in space distribution. The REE concentrations are higher around the mine tailing and decrease with increasing distance away from the central region of the tailing area. Several high REE concentration spots around the tailing region were identified in the estimated map. These findings suggest that the duration of rare earth tailing can significantly contribute to REE accumulation in soils. It also shows a geographical trend, with high REE concentrations in the southeastern area, which might be caused by the strong northwesterly winds in this region.

The calculated EFs for all 14 REEs in soil samples around the tailing region are listed in Table 6. The average EFs of total REEs in east, southeast and south of the tailing were higher than 40, which confirmed an extremely high contamination of REEs in soils in these directions, especially southeast. The average EF of total REEs in the northwest was classified as significant enrichment with an average EF lower than 20. The average EF of total REEs in other directions ranged between 20 and 40, indicating a very high enrichment. For a single REE, EFs can be used to differentiate a natural origin from anthropogenic sources in this study. For instance, mean values of EFs for La, Ce and Pr in east, southeast and south directions were much higher than 40, indicating an extremely high contamination. Most of the HREEs, such as Ho, Er, Tm, Yb and Lu, had mean EFs between 2 and 5, reflecting a moderate enrichment of these elements. The EF analysis confirms that REEs are enriched in soil with varying levels, which is caused by tailing sources and influenced by the prevailing wind in this region.

### Vertical distribution of REEs in soil

**Figure 3:**
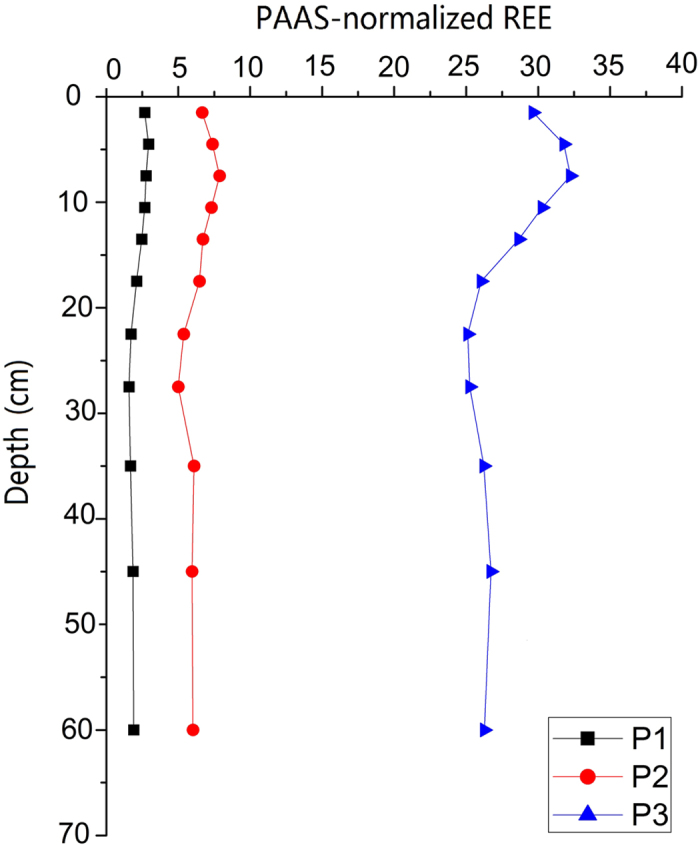
Vertical distributions of ∑REE in the soil profiles normalized to PAAS.

**Figure 4:**
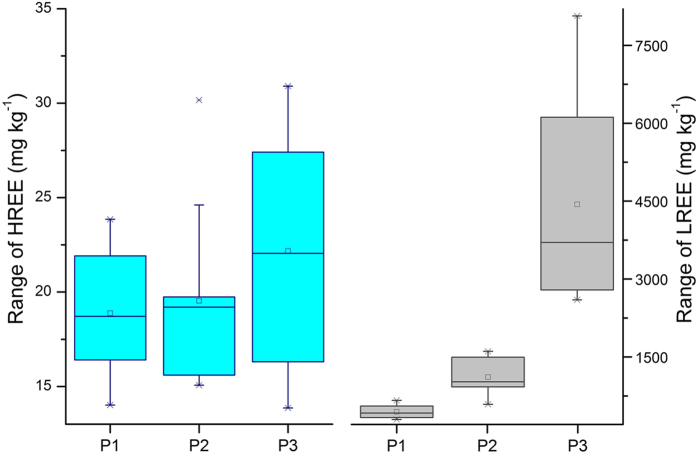
Range of HREE and LREE concentrations in three soil profiles around the mine tailing.

The studied REEs showed similar trends in vertical distribution in the three soil profiles, which further firmed their common chemical and physical properties. The PAAS-normalized REE patterns show clearly that soil samples have elevated total REE contents and similar distribution patterns in the three soil profiles. As shown in Fig. 3, the total REE contents in the profile increased with increasing depth from surface to 9 cm depth, then decreased markedly from 10 to 30 cm depth, and increased slightly below the depth of 30 cm, where the smallest concentrations were observed. Nevertheless, the REE concentrations differed greatly within each soil profile (Fig. 3). The total concentration of REEs in the profile P3 from the south direction was the highest, ranging from 2.62 × 10^3^ to 8.10 × 10^3^ mg·kg^−1^ with an average of 4.46 × 10^3^ mg·kg^−1^ (Figs 3,4). For the profile P1 from the east direction and P2 from the southeast direction, the following measurements were recorded: minimum of 309 and 9.42 × 10^2^ mg·kg^−1^, maximum of 689 and 1.64 × 10^3^ mg·kg^−1^, and mean of 463 and 1.22 × 10^3^ mg·kg^−1^ for P1 and P2, respectively. The REE fractions of these soil profiles also showed an enrichment of LREE relative to the HREE (Fig. 4). These results indicate that the REEs patterns of the soils around the mine tailings are consistent with those of Bayan Obo ores reported in the literature^28^. The obvious enrichment of REEs in the upper parts of the soil profiles may be attributed to the mine tailing pollution and influenced by the soil forming processes. The development of the surface soils is interrupted through frequent flooding events and associated events such as sedimentation and erosion^33^. The REE distributions are influenced by both those processes and the soil physico-chemical properties such as clay content, pH, cation exchange capacity, and organic matter content^34^.

**Figure 5:**
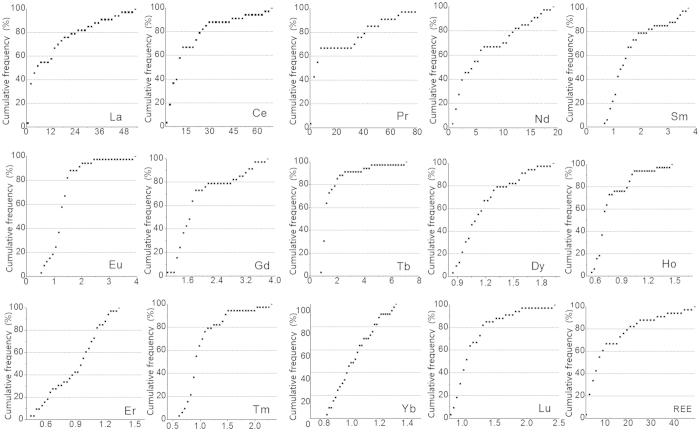
Cumulative frequency distribution of EF of REEs in the soil profiles.

Figure 5 shows the cumulative frequency distributions of REE EFs in the soil around the tailing area. The mean value of EFs for total REEs was 25.4 (ranged from 1.86 to 48.9), indicating a significant enrichment of REEs. The EF values of La, Ce, and Pr in all soil profiles samples were greater than 5, indicating a significant enrichment of these elements. The EF values of Sm and Gd were between 2 and 5, corresponding to a moderate enrichment. The EF values of Ho, Yb, Sm, Tb, Tm, Lu, and Er were less than 2, corresponding to a minimal enrichment. It should be noted that the following elements were not found in all samples, and the percentage of soil samples they were found within are as follows: Gd 55%, Sm 78%, Tb 84%, Tm 94%, and Lu 97%. The enhancement and fluctuation of REEs in soil profiles are mainly attributed to the historical pollution from tailing and anthropogenic disturbance.

### REE differentiation patterns

**Figure 6:**
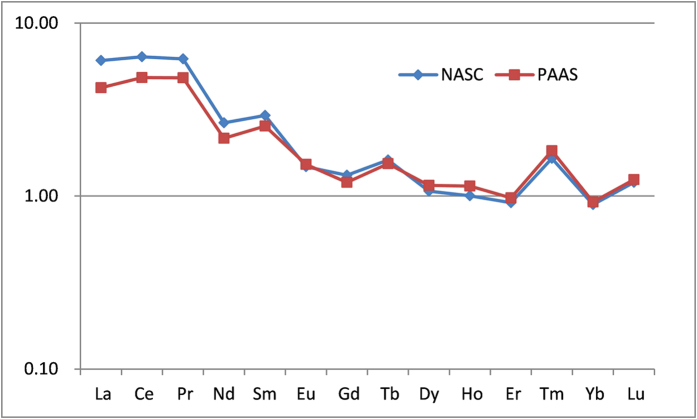
NASC and PAAS normalized patterns of average REE concentrations in surface soils.

To eliminate the Oddo-Harkins effect and characterize the REE signature of soil, the concentrations of individual REEs were normalized to the estimated average composition of REEs in the North American Shale Composite (NASC) and Post-Archean Average Australian Shale (PAAS)^32^, as shown in Fig. 6. The two shale-normalized REE distribution patterns for soil were generally identical, indicating the consistency of geochemical distribution of REEs in every shale. However, the REE patterns, the curves extending downward from left to right, were characterized by LREE enrichment and HREE depletion. The ratio of La_N_/Yb_N_ quantifies the inclination of the shale-normalized curves. When the ratio of La_N_/Yb_N_ is greater than or equal to 1, the curves of LREE incline to right side, meaning that the soil is rich in LREE and low in HREE. The ratio of La_N_/Yb_N_ was 6.80_NASC_ and 4.59_PAAS_, meaning that the soil samples around the rare earth mine tailing belong to a LREE soil type intensified by the pollution. Generally, higher concentrations of LREE are observed in soils that developed on phosphate and carbonate rocks, whereas the basalt-weathered soils show enrichment in HREE^35^. The normalized LREE_N_/HREE_N_ ratio was 5.01_NASC_ and 3.75_PAAS_. The ratios of La_N_/Sm_N_ and Gd_N_/Yb_N_ were used to measure the degree of LREE and HREE, respectively. The degree of LREE fractionation, La_N_/Sm_N_ of 2.08_NASC_ and 1.67_PAAS,_ was slightly higher than that of HREE fractionation, (Gd/Yb)_N_ of 1.48_NASC_ and 1.30_PAAS_.

The depletion or enrichment of Ce and Eu usually occurs in the natural environment, which may be linked to their oxidation state and mobility under different oxidation-reduction conditions^36^. Ce of both oxidation states are found in soils, but under redox conditions. Ce^3+^ is more easily oxidized to Ce^4+^ with higher oxygen fugacity and is much less mobile resulting in positive Ce anomaly (*δ*Ce > 1). Eu is an incompatible element in the trivalent form (Eu^3+^) in an oxidizing magma, but is preferentially incorporated into plagioclase in its divalent form (Eu^2+^) in a reducing magma. This ion-exchange process is the basis of the negative Eu anomaly (*δ*Eu < 1). A slightly positive Ce anomaly, δCe of 1.04_NASC_ and 1.07_PAAS_, and a slightly negative Eu anomaly, δEu of 0.70_NASC_ and 0.82_PAAS_, were also observed, indicating that differentiation occurred between Ce, Eu, and other REEs in weathering process of parent rock.

**Figure 7:**
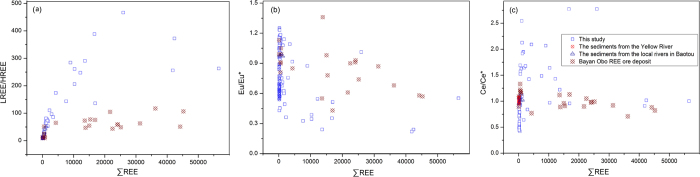
Cross-correlation plots of various geochemical parameters for REEs.

The properties of REE composition and differentiation patterns are not influenced by weathering, transportation process, sedimentation and diagenesis. Meanwhile the provenance information carried by REE remains essentially unchanged, and consequently REEs could be used as a provenance indicator in the geochemical studies^37^. The binary cross-correlation plots of various geochemical parameters were used to discriminate natural variation in REE concentrations from other sources which would influence REE levels. REEs data in recent literatures for 38 fluvial sediments from the Yellow River^38^, 5 sediment samples from the local rivers in the Baotou city^39^ and 24 samples from the Bayan Obo REE deposit^40^ were selected to assess REE enrichment originating from anthropogenic input. Combined with the REEs data from the literatures, the cross plots between total REEs and REEs differentiation characteristics, such as LREE/HREE ratio, Eu anomaly and Ce anomaly were shown in the Fig. 7. The geochemical parameters of REE composition from the present study exhibited a wide range of variability. The surface soil samples around the REE tailings displayed high LREE/HREE ratios, distinct negative Eu anomalies and pronounced positive Ce anomalies when compared with the fluvial sediments and REE minerals, which influenced by the original source of REEs and significantly enhanced by the bare tailings, whose powders were easily dispersed by the strong wind in this area. It has been demonstrated that fine grain size may contribute to the enrichment of the REE abundance^37^,^41^. There have also been reports suggesting that a positive correlation existed between the REE differentiation and the gradation of the dust grain size^41^. These results confirmed that the REE distribution characters reflected the compositions of materials in the source regions, but are also influenced by weathering and pedogenesis.

## Conclusions

Influenced by the mining of rare earth minerals, REEs in surface soils around the Bayan Obo deposit showed different degrees of enrichment. The total content of REEs ranged from 156 to 5.65 × 10^4^ mg·kg^−1^ with an average value of 4.67 × 10^3^ mg·kg^−1^. The order of the average concentrations of REEs in surface soils around the Bayan Obo mine region was as follows (in decreasing order): Ce > La > Nd > Pr > Sm > Gd > Dy > Er > Yb > Eu > Tb > Ho > Tm > Lu, which was similar to that found in Bayan Obo ores, suggesting that the concentration and distribution of REEs are influenced by the mine tailing. The spatial variability of REE concentrations in soil was high in the mine tailing area, which might be caused by human activities and the strong northwesterly winds in this region. The total REE concentration in soil profiles increased with increasing depth from the surface to 9 cm, decreased markedly from 10 to 30 cm, and then increased slightly below the depth of 30 cm, where the minimum concentrations were observed. The concentrations of individual REEs in soil profiles showed a similar trend with depth as that of the total REEs. The degree of LREE fractionation with La_N_/Sm_N_ of 2.08_NASC_ and 1.67_PAAS_ was slightly higher than that of HREE fractionation with (Gd/Yb)_N_ of 1.48_NASC_ and 1.30_PAAS_. Slightly positive Ce anomaly with δCe of 1.04_NASC_ and 1.07_PAAS_, and slightly negative Eu anomaly with δEu of 0.70_NASC_ and 0.82_PAAS_ were also observed.

## Additional Information

**How to cite this article**: Wang, L. and Liang, T. Geochemical fractions of rare earth elements in soil around a mine tailing in Baotou, China. *Sci. Rep.* **5**, 12483; doi: 10.1038/srep12483 (2015).

## Figures and Tables

**Table 1:** Compositional analysis of the Baotou rare earth tailings (wt.%).

Chemical Compositions	TFe	Nb_2_O_5_	CaO	MgO	MnO	SiO_2_	BaO	Na_2_O	CaF_2_	K_2_O	REO	LOI*	Others
	22.21	0.19	22.87	2.82	1.02	24.43	3.03	1.22	10.21	0.68	6.48	2.96	1.88
Mineral Compositions	Magnetite	Hematite	Bastnaesite	Monazite	Fluorite	Carbonate Minerals	Apatite	Barite	Quartz Feldspar	Hornblende Pyroxene	Biotite	Others	
	2.71	21.55	6.12	2.66	21.12	4.72	6.39	1.78	8.28	17.63	4.11	2.93	

^*^LOI: loss on ignition at 1000 °C.

**Table 2:** Selected properties of soil in Baotou district.

Organic matter/%/%	pH	Electrical conductivity/μs·cm^−1^	TP	Olsen P	TN/%	Soil particle size (%)		
			mg·kg^−1^			Sand	Silt	Clay
1.78	7.33	112.52	957.42	33.95	0.11	48.06	39.25	12.69

**Table 3:** Descriptive statistics of REEs concentration in surface soil (mg/kg).

	Mean	SD	CV (%)	Min	Max	GM	K-S	Background value	UCC
La	891.59	2237.82	251.0	42.52	11941.46	188.79	<0.001	32.8	30
Ce	2954.47	6418.93	217.3	55.51	31736.44	427.59	<0.001	49.1	64
Pr	355.20	908.96	255.9	8.05	4881.33	49.05	<0.001	5.68	7.1
Nd	384.44	1193.90	310.6	12.79	7226.95	80.62	<0.001	19.2	26
Sm	48.16	99.83	207.3	4.01	521.30	17.47	<0.001	3.81	4.5
Eu	2.76	3.65	132.6	0.52	21.74	1.85	<0.001	0.81	0.88
Gd	10.30	14.05	136.5	3.48	83.81	7.25	<0.001	3.47	3.8
Tb	2.50	4.89	195.6	0.58	31.61	1.37	<0.001	0.47	0.64
Dy	7.85	8.79	112.0	2.52	56.69	6.13	<0.001	3.05	3.5
Ho	2.06	3.37	163.4	0.50	13.97	1.20	<0.001	0.66	0.8
Er	3.19	1.38	43.3	1.56	9.09	3.00	<0.001	1.82	2.3
Tm	1.36	1.69	123.7	0.25	8.35	0.83	<0.001	0.27	0.33
Yb	2.90	0.96	33.1	1.99	7.17	2.78	<0.001	1.79	2.2
Lu	0.69	0.61	89.1	0.27	3.33	0.55	<0.001	0.27	0.32
∑REE	4667.47	10619.54	227.5	155.65	56543.2	883.44	<0.001	123.2	146.37
LREE	4636.62	10587.62	228.3	141.52	56329.22	841.44	<0.001	111.4	132.48
HREE	30.85	34.01	110.3	12.83	214.01	24.15	<0.001	11.8	13.89

SD, standard deviation; CV, coefficient of variation; GM, geometric mean.

**Table 4:** Pearson’s correlations between REE concentrations in the surface soil samples (n = 67, p < 0.05).

	La	Ce	Pr	Nd	Sm	Eu	Gd	Tb	Dy	Ho	Er	Tm	Yb	Lu
La	1													
Ce	0.92	1												
Pr	0.91	0.95	1											
Nd	0.92	0.95	0.75	1										
Sm	0.89	0.89	0.92	0.92	1									
Eu	0.77	0.75	0.73	0.73	0.82	1								
Gd	0.73	0.73	0.84	0.88	0.68	0.78	1							
Tb	0.59	0.63	0.65	0.82	0.73	0.75	0.94	1						
Dy	0.52	0.58	0.63	0.58	0.69	0.58	0.92	0.83	1					
Ho	0.33	0.61	0.52	0.65	0.75	0.67	0.93	0.91	0.91	1				
Er	0.42	0.62	0.57	0.68	0.62	0.86	0.61	0.78	0.82	0.78	1			
Tm	0.25	0.33	0.48	0.65	0.73	0.65	0.76	0.82	0.72	0.78	0.81	1		
Yb	0.37	0.58	0.33	0.54	0.83	0.58	0.82	0.78	0.77	0.82	0.96	0.88	1	
Lu	0.36	0.62	0.48	0.32	0.49	0.73	0.73	0.79	0.79	0.62	0.95	0.79	0.91	1

**Table 5:** Parameters of fitted variogram model for REEs in surface soils.

Variogram model	Nugget C_0_ (m)	C (m)	C_0_/C_0_+C	Spatial range (m)	R^2^
Spherical	100000	160900000	0.000622	4930	0.68

**Table 6:** EFs of REEs in surface soil samples around the mine tailing region.

REEs	East (n = 10)	Northeast (n = 9)	North (n = 6)	Northwest (n = 7)	West (n = 8)	Southwest (n = 7)	South (n = 9)	Southeast (n = 11)
La	57.85	32.15	20.39	22.98	26.51	50.44	68.49	79.03
Ce	88.84	27.88	38.73	26.17	33.16	42.47	129.55	153.64
Pr	119.48	21.27	25.21	33.32	29.40	39.47	141.36	166.86
Nd	33.55	8.59	7.53	8.47	8.43	14.27	41.70	48.46
Sm	22.44	12.16	9.83	8.97	7.78	7.88	27.52	30.22
Eu	3.50	4.30	3.00	3.00	2.71	4.63	3.33	12.79
Gd	4.01	3.55	3.29	3.25	3.15	6.37	4.72	5.92
Tb	6.36	3.44	2.87	3.03	2.41	4.24	9.56	13.88
Dy	3.89	2.86	2.83	3.09	3.04	3.31	4.58	4.51
Ho	4.38	4.38	5.50	3.74	1.96	5.06	4.31	4.15
Er	2.26	2.26	2.23	2.52	2.00	2.41	2.12	2.16
Tm	5.99	2.77	3.19	3.37	3.33	5.48	11.47	13.57
Yb	2.02	1.85	1.95	2.65	2.07	2.10	2.04	2.29
Lu	3.66	2.83	2.99	2.45	2.70	4.15	3.45	4.78
∑REE	63.51	22.01	24.27	19.85	23.37	34.01	86.21	101.54
